# Direct Foliar Application of dsRNA Derived From the Full-Length Gene of *NSs* of Groundnut Bud Necrosis Virus Limits Virus Accumulation and Symptom Expression

**DOI:** 10.3389/fpls.2021.734618

**Published:** 2021-12-07

**Authors:** Dipinte Gupta, Oinam Washington Singh, Y. B. Basavaraj, Anirban Roy, Sunil Kumar Mukherjee, Bikash Mandal

**Affiliations:** Division of Plant Pathology, Advanced Centre for Plant Virology, Indian Agricultural Research Institute, New Delhi, India

**Keywords:** groundnut bud necrosis virus, GBNV, tospovirus, dsRNA mediated protection, topical application of dsRNA, *NSs* gene

## Abstract

Groundnut bud necrosis virus (GBNV) is the most significant member of the genus *Orthotospovirus* occurring in the Indian subcontinent. There is hardly any effective measure to prevent GBNV in crop plants. In order to develop GBNV infection prevention procedure, we examined the effect of the direct foliar application of double-stranded RNA (dsRNA) derived from the full-length *NSs* gene (1,320 nucleotides) of GBNV. The bacterially expressed dsRNA to the non-structural (dsNSs) gene of GBNV was purified and delivered to plants as an aqueous suspension containing 0.01% Celite for evaluating its efficacy in preventing GBNV infection in systemic host, *Nicotiana benthamiana* as well as in local lesion and systemic host, cowpea cv. Pusa Komal (*Vigna unguiculata*). The dsNSs application and challenge-inoculation were conducted in three different combinations, where plants were challenge-inoculated with GBNV a day after, immediately, and a day before the application of dsNSs. *N. benthamiana* plants, which were not treated with dsRNA showed severe systemic wilting and death by 9–16 days post-inoculation (dpi). The non-treated cowpea plants exhibited many chlorotic and necrotic lesions on the cotyledonary leaves followed by systemic necrosis and death of the plants by 14–16 dpi. The dsNSs treated plants in all the combinations showed significant reduction of disease severity index in both *N. benthamiana* and cowpea. The treatment combination where the GBNV inoculation was conducted immediately after the dsNSs treatment was found to be the most effective treatment in preventing symptom expression. The viral RNA analysis by real time PCR also showed 20 and 12.5 fold reduction of GBNV in cowpea and *N. benthamiana*, respectively. Our results suggest that the foliar application of dsRNA derived from the full-length *NSs* gene of GBNV through Celite is successful in delivering long dsRNA leading to effective prevention of GBNV infection.

## Introduction

Negative-sense ssRNA plant viruses are classified within the four families, *Ophioviridae, Phenuiviridae, Tospoviridae*, and *Rhabdoviridae*. Members of family, *Tospoviridae* are highly significant viruses as they are known to infect >1,000 plant species and are distributed all over the world (Kormelink et al., 2011). The genome of orthotospoviruses contains three segments of ssRNA: large (L), medium (M), and small (S) (Pappu et al., 2009). The L RNA is of negative polarity and encodes for the RNA-dependent RNA polymerase. The M RNA contains two genes, one in positive sense orientation encoding the movement protein and the other in negative sense orientation encoding Gn and Gc proteins required for thrips transmission. Similarly, the S RNA also contains two genes, the sense oriented one encodes a non-structural (NSs) protein that plays the role of a suppressor of RNA silencing and symptom determinant, and the antisense-oriented gene encodes for the nucleoprotein (NP) (Prins and Goldbach, 1998; Akram et al., 2004). Orthotospoviruses are transmitted by thrips vectors in a persistent propagative manner and are serious viral pathogens of numerous plant species. There are 26 orthotospovirus species^1^ known all over the world. Tomato-spotted wilt virus (TSWV) is the most widespread and most studied tospovirus species (Pappu et al., 2009), however, in the Indian subcontinent, groundnut bud necrosis virus (GBNV) is the most prevalent orthotospovirus species. The complete genome sequence of GBNV has been generated and limited gene function studies have been conducted (Basavaraj et al., 2017). The NSs protein of GBNV has been shown as a bifunctional enzyme having both ATPase and phosphatase activities (Lokesh et al., 2010). Subsequently, the NSs protein of GBNV was found to be the symptom determinant, suppressor of RNA silencing, and inducer of plant cell death (Goswami et al., 2012; Singh et al., 2017). GBNV infects a wide variety of important crops such as cowpea, mungbean, peanut, potato, tomato, soybean, and urdbean. The conventional management approaches with cultural practices and insecticide treatment are not much effective to manage GBNV due to its wide host ranges, the abundance of thrips vector, and the lack of resistance cultivars which lead to a frequent disease outbreak causing serious crop losses (70–90%) in India (Reddy et al., 1995; Mandal et al., 2012). A few studies have attempted to utilize the molecular approaches to prevent GBNV infections, where the sense or antisense, hairpin, and artificial micro RNA constructs derived from *NP* and *NSs* genes of GBNV were shown to reduce GBNV infection in the experimental transgenic plants like tobacco and cowpea (Venkatesan et al., 2009; Goswami et al., 2012; Babu et al., 2019).

RNA interference (RNAi) plays a significant role in the plant development and defense against invasive nucleic acid such as transposable elements, virus, and virus-like pathogens (Agrawal et al., 2003; Obbard et al., 2009). For silencing or knocking down the expression of specific viral genes, plants have developed a strategy known as post-transcriptional gene silencing (PTGS) that involves sequence-specific degradation of viral RNA conferring resistance to plants (Unver and Budak, 2009). The important feature of this mechanism is to process the double-stranded RNA (dsRNA) or partial overlapping transcripts of DNA viruses into small interfering RNA (siRNA) of approximately 21–24 nucleotides by the enzymatic activity of dicer like enzymes (Carthew and Sontheimer, 2009). The processed siRNA binds to argonaute (AGO) protein and then is incorporated into RNA-induced silencing complex, which ensures degradation of the target RNA or viral transcript sharing the sequence similarity with the siRNA (Bologna and Voinnet, 2014). Additionally, the complementary guide RNA can also serve as a primer for the RNA-dependent RNA (RDR) polymerase for the generation of secondary siRNA ensuring amplification of siRNA signal. The RNAi-based defense mechanism is being utilized as a powerful strategy to develop disease resistance in crop plants against pests and pathogens such as viruses (Sanan-Mishra et al., 2021).

The principle of RNAi has been extensively utilized for engineering transgenic resistance in plants against viruses through genetic modification of plants with a segment of nucleotide sequence from the viral genome. The transgenic technology has been successfully utilized to develop virus-resistant cultivars of different crop plants (Leibman et al., 2011; Zhao et al., 2019; Gaffar and Koch, 2019). However, the practical field application of transgenic technology is greatly limited by the stringent regulatory laws in the different countries. An alternative approach to the transgenic technology is to induce RNAi in plants against the viruses through external or topical application of dsRNA derived from the viral genome. Topical application of dsRNA is emerging as an appealing non-GMO approach for the effective management of plants against virus infection (Dubrovina and Kiselev, 2019; Taliansky et al., 2021). To date, the studies on the topical application of dsRNA have been conducted against at least 14 plant viruses having positive sense ssRNA as their genome (Taliansky et al., 2021). To some extent, the approach was also found to be effective against ssDNA virus (genus *Begomovirus*), however, not much information is available as only three studies are available that showed limited or no effect against *Begomovirus* (Namgiala et al., 2019; Rego-Machado et al., 2020; Melita et al., 2021). Recently, two studies had shown the efficacy of topically applied dsRNA against the type member of the genus *Orthotospovirus*, TSWV, a negative/ambisense ssRNA virus (Tabein et al., 2020; Konakalla et al., 2021). However, no study so far demonstrated the usefulness of the exogenous application of dsRNA against GBNV.

The objective of the present study was to examine the potentiality of foliar application of dsRNA derived from the full-length gene of *NSs* (dsNSs) of GBNV in the two hosts, *N. benthamiana*, a systemic host and *Vigna unguiculata* (cowpea), a local lesion and systemic host. Our study demonstrated that the foliar application of dsNSs significantly reduced symptom expression as well as the viral load in both systemic and local host plants indicating high potential for developing anti-tospoviral RNA-therapeutic.

## Materials and Methods

### Test Plant and Virus Isolate

Seedlings of *N. benthamiana* and *V. unguiculata* cv. Pusa Komal (cowpea) were raised in potting-mix in 10 cm pots in an environmentally controlled growth room with an average temperature of 24 ± 2°C, average humidity of 65% and 16/8 h of light and dark periods. GBNV was isolated from a tomato plant showing leaf and bud necrosis, which was confirmed by ELISA with the polyclonal antibody to GBNV and subsequently sequencing the *NP* gene. The virus culture was maintained in both cowpea and tomato host through sap inoculation.

### Preparation of dsRNA

A full-length *NSs* gene was amplified by RT-PCR from the above GBNV culture and cloned into pGEM T-Easy vector. The sequence of NSs was submitted to the GenBank with the accession number, OK105104. The *NSs* gene was further sub-cloned in the L4440 vector^2^ having a T7 promoter at both ends. The recombinant vector was then used to transform the *Escherichia coli* strain HT115 for the production of dsRNA. To optimize the conditions for dsRNA generation, experiments with the different concentrations of IPTG (0.01, 0.1, 0.4, and 1.0 mM), different induction period after IPTG treatment (30 min, 1 h, 4 h, and overnight) and with different enzyme treatments [RNase A alone in buffer containing 300 mM sodium acetate, 10 mM Tris–HCl pH 7.5 and 5 mM EDTA (Thermo Fisher Scientific, United States) or without buffer; DNase I alone in buffer containing 10 mM Tris–HCl pH 7.5, 2.5 mM MgCl_2_, 0.5 mM CaCl_2_ (New England Biolabs, United Kingdom); both RNase A and DNase I together and without any enzyme treatment] were evaluated and the best combination of these factors were utilized for the production of dsRNA.

Purification of dsRNA from the bacterial culture was followed with some modification of the protocol as described by Posiri et al., 2013. Briefly, the IPTG-induced bacterial cells from 100 ml culture were harvested and used for the purification of dsRNA. The cell pellet was re-suspended in 1X PBS containing 70% v/v ethanol and incubated at 4°C for 30 min. After incubation, the cell pellets were collected by centrifugation at 6,000 × *g* for 10 min at 4°C. Then, the bacterial cells were re-suspended in 2.0 ml of 150 mM NaCl and incubated at 4°C for 1 h and centrifuged at 6,000 × *g* for 10 min at 4°C. To the supernatant, four units of RNase free DNase I and 20 μg of RNase A were added at 37°C for 30 min to remove DNA and ssRNA. The dsRNA was then precipitated by adding 500 μl of absolute ethanol. The pellet was air-dried, dissolved in deionized water, and the quantity was estimated spectrophotometrically (NanoDrop, Thermo Fisher Scientific, United States). The dsRNA preparation was stored at −20°C for further use. The quality of the purified dsRNA was judged by visualizing the expected dsRNA band on agarose gel. To examine the double stranded nature of the purified preparation, RNase treatment was performed in high and low salt concentration (Libonati and Sorrentino, 1992). Further, to confirm that the obtained band was of dsRNA from the *NSs* gene, nucleic acid hybridization assay (Northern/dot blot) was conducted with a DIG-labeled probe to the full-length *NSs* gene, which was prepared using DIG-High Prime DNA Labeling and Detection Starter Kit I (Roche, F. Hoffmann-La Roche Ltd., Switzerland).

### Delivery of dsRNA to Plant

In order to know, the entry of dsRNA into the plant system, the dsRNA aqueous solution containing 0.01% Celite 545 (BDH, England) was applied at the rate of 5.0 μg of dsRNA/plant by gentle rubbing on the adaxial surface of cowpea and *N. benthamiana* leaves. The excess dsRNA present on the leaf surface was removed by washing 4–5 times with distilled water. To detect the dsRNA in the different washes, each washout-water was collected separately, lyophilized to approximately 20 μl volume, and loaded in 1.5% agarose gel. The dsRNA treated leaves, after thorough and repeated washing, were used to isolate RNA by using TRIzol (Thermo Fisher Scientific)^3^. To understand the stability of the entered dsNSs in the plant tissues, cDNA of *NSs* was prepared using the total RNA isolated from the treated leaves with the reverse primer of *NSs* (BM1251: 5′ataagcttttactctggcttcacaatga3′) using Revert Aid First Strand cDNA Synthesis Kit (Thermo Fisher Scientific, Waltham, MA, United States). The stability pattern of dsNSs in the plant tissue was analyzed in the local leaves (treated) of cowpea as well as both the local and systemic (non-treated) leaves of *N. benthamiana* by semi-quantitative reverse transcriptase PCR (semi-qRT-PCR) with the primer pair, BM1260: 5′gacagatgcagagggaaatg3′; BM1251: 5′ataagcttttactctggcttcacaatga3′ at 15, 28, and 35 PCR cycles. The PCR conditions were: initial denaturation at 95°C for 3 min, followed by each cycle of denaturation at 95°C for 30 s, annealing at 52°C for 30 s, and extension at 72°C for 30 s. The final extension at 72°C was allowed for 5 min. The *GAPDH* and *actin* genes (Reddy et al., 2016) were used as internal controls for cowpea and *N. benthamiana*, respectively.

### Challenge-Inoculation With Groundnut Bud Necrosis Virus

The challenge-inoculation of dsNSs-treated plants with GBNV was performed as described by Rai et al., 2020. The GBNV inoculated *N. benthamiana* leaves showing initiation of mottling and bending symptoms were used to prepare inoculum in the extraction buffer at a ratio 1:6 (1.0 g of leaves in 6 ml of extraction buffer) in a pre-chilled mortar and pestle. The extraction buffer was composed of 0.1 M sodium phosphate buffer, pH 7.0, containing 0.15% sodium sulfite, and 0.002% beta-mercaptoethanol. The test plants were pre-dusted lightly with Celite powder and then 100 μl of the inoculum was applied gently on the surface of each leaves. The inoculated plants were sprayed with distilled water and maintained in a controlled environment room for symptom expression.

### Treatments for Assessing dsNSs Against Groundnut Bud Necrosis Virus

The effectiveness of dsNSs against GBNV was assayed in three different treatment combinations. (T1) dsNSs treatment followed by GBNV (dsNSs – GBNV): in this combination, 5.0 μg of dsNSs dissolved in deionized water containing 0.01% Celite was applied by gentle rubbing on the adaxial surface of leaves and 1-day post-application of dsRNA, plants were challenged with GBNV through mechanical sap inoculation by the method as described above. (T2) Succeeding application of dsNSs and GBNV (dsRNA + GBNV): in this combination, 5.0 μg of dsNSs solution containing 0.01% Celite was similarly applied on the leaves and allowed for 10–15 min and then plants were challenged by mechanical sap inoculation with GBNV. (T3) GBNV inoculation followed by dsNSs treatment (GBNV – dsNSs): in this combination, leaves were first sap inoculated with GBNV, and at 1-day post-inoculation (dpi), 5.0 μg of dsNSs solution containing 0.01% Celite was applied. For the above experiment, two fully expanded primary leaves of cowpea and two leaves of 1-month-old *N. benthamiana* plants were used for treating with dsRNA and GBNV inoculation. The experiment with these three treatment combinations was repeated three times on *N. benthamiana* and cowpea var. Pusa Komal. The experimental plants were maintained for further observations and the number of chlorotic and necrotic lesions in local and systemic leaves were counted at regular intervals in cowpea and the systemic disease progress was monitored for both *N. benthamiana* and cowpea.

### Disease Severity Analysis

To assess the impact of dsNSs treatment on symptom expression by GBNV, disease rating scales were developed on the basis of symptom severity grades by assigning the numerical values of 0–4 and 0–8 for *N. benthamiana* and cowpea, respectively (Table 1). The disease severity index (DSI) was assessed based on the disease incidence, which was calculated as [Σ (Grade assigned × number of infected plants)/Total grade × total number of plants] × 100. The relative progression of the disease till the death of the dsNSs-untreated GBNV inoculated plants was recorded and calculated using the area under the disease severity curve (AUDSC), following the standard method (Campbell and Madden, 1990; Bag et al., 2014) with the formula $\text{Y}=\sum_{i=1}^{n-1}\left[\left({\text{X}}_{\text{i}}+{\text{X}}_{\text{i}+1\,}\right)/2\right]$(t_i+ 1_−t_i_) where Y is the AUDPC, X_i_ is the disease incidence of the i^th^ evaluation and X_i+1_ is the disease incidence of the subsequent evaluation; (t_i+1_-t_i_) is the number of days between two subsequent evaluations.

**TABLE 1:** Disease grading scales of groundnut bud necrosis virus in cowpea and *Nicotiana benthamiana.*

Cowpea		*N. benthamiana*	
Symptom	Grade	Symptom	Grade
No symptom	0	No Symptom	0
Chlorotic patches	1	Chlorotic/necrotic lesions (1–5)	1
Bending of shoots	2	Chlorotic/necrotic lesions (5–15)	2
Wilting of leaves	3	Chlorotic/necrotic lesions (15–30)	3
Death of plant	4	Complete leaf necrosis	4
		Wilting and leaf fall	5
		Systemic necrosis on new leaves	6
		Petiole and stem necrosis	7
		Death of plant	8

### Quantification of Viral Load in the dsNSs-Treated Plants

The viral load with or without dsNSs treatment was analyzed using semi qRT-PCR as well as qPCR. On the onset of disease symptoms at 9 dpi in *N. benthamiana* and at 6 dpi in cowpea, RNA was isolated from the fresh leaf samples. cDNA was prepared with 1.0 μg of RNA sample and random hexamers by using Revert Aid First Strand cDNA Synthesis Kit (Thermo Fisher Scientific, Waltham, MA, United States). Semi qPCR was performed using 1.0 μl of cDNA as a template. For performing qPCR analysis, 1.0 μl of cDNA was used as template and a portion of *NP* gene of GBNV was amplified using specific primers (PF: 5′GACAGGTCTGGCACCAATTA3′ and PR: 5′GGCTACTTTGCAAACCTGTTC3′) with 1× KAPA SYBR^®^ FAST qPCR Master Mix (Roche, F. Hoffmann-La Roche Ltd., Switzerland). The *actin* and *GAPDH* genes were used as an endogenous control gene for *N. benthamiana* and cowpea, respectively. The relative viral load in the dsNSs-treated plants was calculated using the double C_t_ method (Livak and Schmittgen, 2001) comparing with the viral load as 1 unit in the untreated plants (calibrator). Three technical replicates were used in all the qPCR analysis.

### Statistical Analysis

For comparison of disease severity between the three combinations of dsRNA with GBNV treatments and the control (only GBNV inoculation), one-way ANOVA test was performed. Homogeneity of variance test (Levene’s statistic) was performed using SPSS package 20.0 to check the equality of variance in three datasets as the experiments were executed independently. Differences in the variance of the treatments were analyzed by Brown–Forsythe statistics.

For qPCR analysis, all the treatment combinations were evaluated as independent experiments and the data were statistically analyzed by ANOVA considering *p*-value < 0.05 as significant. The significance of the data within the dataset is also analyzed by performing student’s *t*-test at *p* < 0.1 and *p* < 0.05 in *N. benthamiana* and cowpea, respectively.

## Results

### dsRNA Derived From the *NSs* Gene of Groundnut Bud Necrosis Virus

**FIGURE 1:**
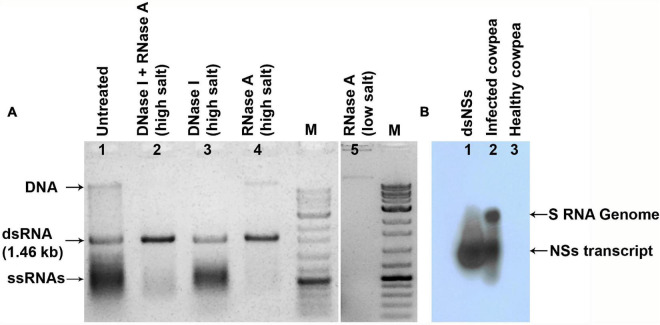
The dsRNA specific to *NSs* gene of groundnut bud necrosis virus (GBNV) expressed in *E. coli* strain HT119. **(A)** Agarose gel showing nuclease digestion of the isolated nucleic acid from the bacteria transformed with pL4440, containing *NSs.* RNase A in high salt resisted digestion of 1.46 kb fragment (lane 4), but completely digested (lane 5) in low salt condition, indicating double stranded form. **(B)** Northern blot sowing hybridization of dsNSs isolated from bacteria (lane 1) and GBNV infected cowpea plant (lane 2) with the DIG-labeled probe to the PCR product of *NSs* gene of GBNV.

The clone of *NSs* gene contained 1,320 nucleotides and shared up to 97.5% sequence identity with that of other isolates of GBNV available in the GenBank. The clone carrying dsNSs in L4440 vector was subjected to the various parameters for the expression of dsRNA in the bacterial culture, which revealed incubation of bacterial culture with 0.4 mM IPTG at 37°C for 4 h induced maximum expression of the expected approximately 1.46 kb dsNSs. Purification of dsRNA from bacteria yielded approximately 1.5–2.0 mg of dsNSs per liter of bacterial culture. The dsRNA from the bacterial cells when was treated with RNase A in high salt, the approximately 1.46 kb band was not digested; however, it was digested with RNase A in low salt condition indicating double stranded nature of 1.46 kb band (Figure 1A). Further, the approximately 1.46 kb band was of *NSs* gene origin was confirmed through both Northern blot as well as dot blot analyses, which showed strong hybridization signal with the DIG-labeled probe to the NSs clone (Figure 1B).

### Entry and Stability of dsNSs in Plants

**FIGURE 2:**
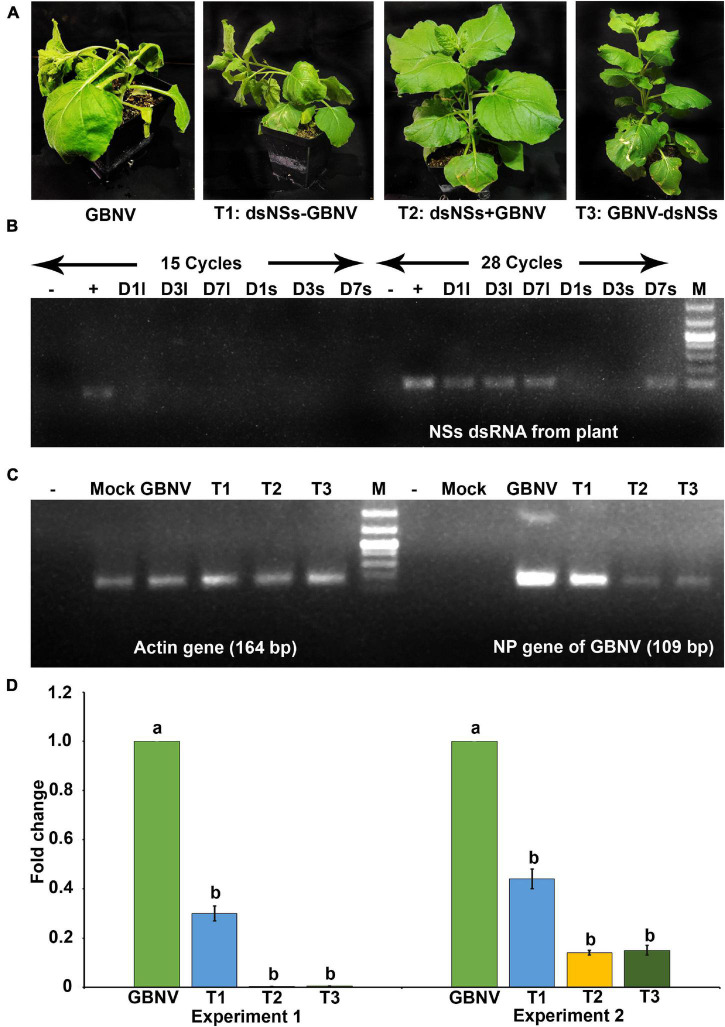
Effect of topically applied dsNSs of GBNV in the *Nicotiana benthamiana* challenged with GBNV at 9 days post inoculation (dpi). **(A)** Treatment combinations: T2: dsNSs + GBNV (dsNSs treatment followed by virus inoculation) and T3: GBNV-dsNSs (dsNSs was applied a day post inoculation with GBNV) showed no necrosis and wilting while treatment combination T1: dsNSs – GBNV (dsNSs was applied a day before GBNV inoculation) as well as dsNSs untreated control plants exhibited those symptoms. **(B)** Stability analysis of dsNSs in the absence of GBNV in the local (l) and systemic (s) leaves during 1–7 dpi (D1–D7) by semiquantitative reverse transcriptase PCR (sqRT-PCR). **(C)** Detection of GBNV in the dsNSs-treated plant by sqRT-PCR of *NP* gene showing reduction of amplification of *NP* gene in different treatments compared to dsNSs untreated control plants. Actin was taken as internal control. **(D)** Quantitative reverse transcriptase PCR (qRT-PCR) showing significant reduction (*p* ≤ 0.05) of GBNV titer in all the three treatment combinations. Different letters indicate statistically significant difference (*p* ≤ 0.05). Two independent experiments were analyzed using Actin gene as endogenous reference control. –, reagent control; +, Plasmid of NSs clone (Positive control); Mock, buffer inoculated healthy plant; M, DNA mark 1 kb plus DNA ladder (G-Biosciences, United States).

**FIGURE 3:**
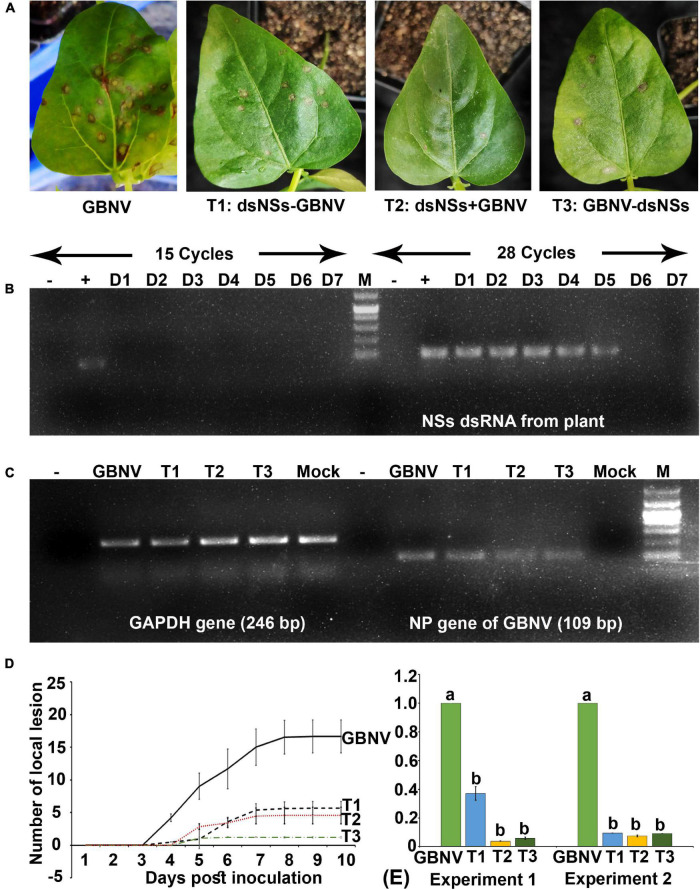
Effect of topically applied dsNSs of GBNV in the cowpea cv. Pusa Komal challenged with GBNV at 6 days post inoculation (dpi). **(A)** The cowpea leaf showing reduced number of local lesions with T1, T2, and T3 treatment combinations compared to dsNSs untreated control plants. **(B)** Stability analysis of dsNSs in the absence of GBNV in the local leaves during 1–7 dpi (D1–D7) by sqRT-PCR. **(C)** Detection of GBNV in the dsNSs-treated plant by sqRT-PCR of *NP* gene. GAPDH was taken as internal control. **(D)** Reduction of the progression of local lesions in the three treatments (T1, T2, and T3) as compared to untreated GBNV inoculated control plants. **(E)** qRT-PCR showing significant reduction (*p* ≤ 0.05) of GBNV titer in all the three treatment combinations. Different letters indicate statistically significant difference (*p* ≤ 0.05). Two independent experiments were analyzed using GAPDH gene as endogenous reference control. –, reagent control; +, Plasmid of NSs clone (Positive control); Mock, buffer inoculated healthy plant; M, DNA mark 1 kb plus DNA ladder (G-Biosciences, United States).

To know the entry of dsRNA in the leaf tissues, the excess dsRNA present on the leaf surface was removed by repeated washing. It was found that after 4–5 washes, no dsRNA on the leaf surface could be detected in the agarose gel electrophoresis. Therefore, while detecting dsRNA in the plant tissue by semi qRT-PCR, the treated leaves were thoroughly washed five times before isolating the total RNA from the plant tissues. In *N. benthamiana* plants, dsNSs was tested by semi qRT-PCR with the NSs primers in the treated as well as in the newly developed leaves at 1, 3, and 7 days post-treatment. At the 15th cycle of semi qPCR, no detection of *NSs* gene was obtained; however, at 28th cycle of semi qRT-PCR, 118 nucleotide fragment of *NSs* was detected at all the three-time points in the treated leaves, interestingly, and NSs could be detected in the systemic leaves at 7 days post-treatment (Figure 2B). In cowpea, NSs was detected at the site of treatment till 5 days post-treatment but not at 6 or 7 days post-treatment at 28 cycles of semi qRT-PCR (Figure 3B).

### Efficacy of dsNSs Against Groundnut Bud Necrosis Virus in *N. benthamiana*

The effectiveness of the topically applied dsNSs of GBNV for the prevention of GBNV infection was tested in 30 days old *N. benthamiana* plants containing 4–5 well-developed leaves (*n* = 4 plants in each of the three experiments). The dsNSs non-treated control plants of *N benthamiana* inoculated with GBNV showed no local lesion on the inoculated site; however, chlorotic patches were observed in these leaves at 9 dpi. Subsequently, systemic symptoms like veinal chlorosis, necrosis of newly developed leaves, and bending of the shoot with wilting of plants from the upper plant part were observed. Finally, the infected plant collapsed and died. To estimate the DSI each of the sequentially advancing symptoms like no symptom, chlorotic patches, bending of the shoot, wilting of leaves, and death of plants were graded as 0, 1, 2, 3, and 4, respectively (Table 1). The GBNV inoculated plants, where dsRNA was not applied, reached the severe disease grade of 3–4 by 9–16 days. Whereas the treatment combination-T2 (dsNSs + GBNV: where the dsRNA application was followed by the virus inoculation immediately), and the treatment combination-T3 (GBNV – dsNSs: where the dsRNA was applied at 1.0 dpi) showed a low disease severity grade of 0–2. The treatment combination-T1: dsNSs – GBNV, where the dsRNA was applied a day before GBNV inoculation, showed higher disease severity compared to that in the treatment combination-T2 and -T3 (Figure 2A). The DSI in the dsRNA-treated plants ranged between 50 and 66.7 indicating a reduction in the disease severity of about 43.3–50% in the dsRNA-treated plants compared to the non-treated controls. The AUDSC showed significantly less AUDSC values (669–924) in the treatment combination-T2 and -T3, where dsRNA treatment was conducted just before GBNV inoculation and a day post-GBNV inoculation, respectively, compared to the non-treated control which showed AUDSC value of 1378 (Table 2). Disease severity in GBNV treatment was significantly higher than the other three treatments (*p* < 0.05) indicated other three treatments were effective against viral infection. Although, homogeneity of variance test was significant (*p* < 0.05), but the differences in the treatments based on Brown–Forsythe statistics as the assumption of equal variances has been violated was found to be significant indicated differences really existed among the treatments.

**TABLE 2:** Effect of topically applied dsRNA of *NSs* gene of groundnut bud necrosis virus (GBNV) on the disease severity in *Nicotiana benthamiana* and cowpea cv. Pusa Komal.

	*N. benthamiana*		Cowpea	
Treatments^1^	DSI^2^	AUDSC	DSI	AUDSC
GBNV	100 ± 0^a^	1378 ± 9^a^	100 ± 0^a^	544.1 ± 26^a^
T1: dsNSs-GBNV	66.7 ± 8.4^b^	994 ± 103.2	84.4 ± 32^b^	427.4 ± 26^b^
T2: dsNSs+GBNV	50 ± 16.7^b^	669 ± 165^b^	9.7 ± 2^b^	86.6 ± 10^b^
T3: GBNV-dsNSs	56 ± 15.4^b^	924.7 ± 161^b^	35 ± 7^b^	188.9 ± 26^b^

*^1^GBNV, Only virus; T1: dsNSs-GBNV, dsNSs was applied on leaves and then a day after, plants were sap inoculated with GBNV; T2: dsNSs+GBNV, dsNSs was applied on leaves, then after 10–15 min, they were sap inoculated with GBNV; T3: GBNV-dsNSs, leaves were inoculated with GBNV and a day after, dsNSs was applied. 5.0 μg of dsNSs was dissolved in autoclaved distilled water containing 0.01% Celite was applied on each leaf.*

*^2^Disease Severity Index (DSI) was at 9 and 14 days post inoculation of Nicotiana benthamiana and cowpea, respectively, as on these days, death of plant started due to GBNV infection in the dsNSs untreated plants. AUDSC, Area under disease severity curve. AUDSC was calculated based on the progression of the disease till the death of the dsNSs untreated GBNV inoculated plants. Three independent experiments were conducted for each treatment. Homogeneity of variance of the experiments was tested by Levene’s statistic using SPSS package 20.0. Differences in the variance of the treatments were analyzed by Brown-Forsythe statistics. Different letters within a column indicate statistically significant difference (p ≤ 0.05).*

**FIGURE 4:**
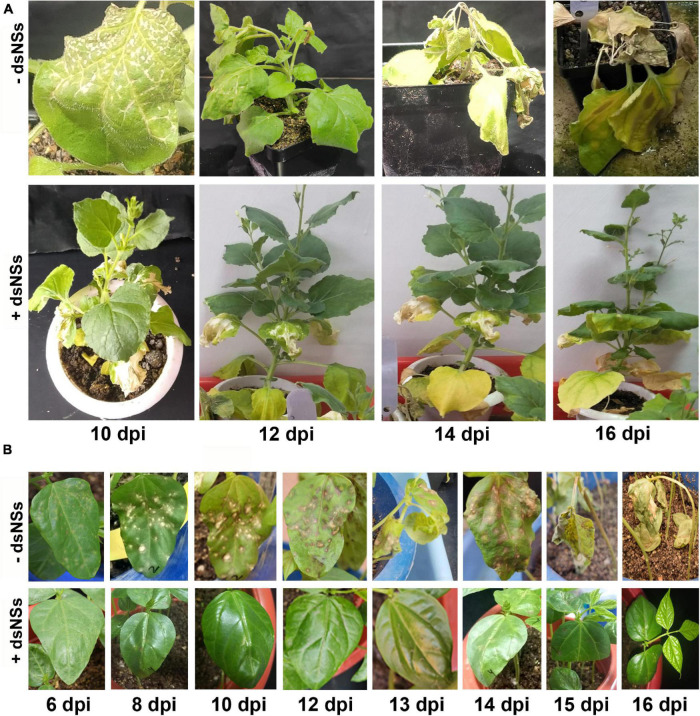
The disease progress of GBNV in *Nicotiana benthamiana* **(A)** and cowpea cv. Pusa Komal **(B)** plants treated with dsNSs. *N. benthamiana* showed systemic symptoms of GBNV, whereas cowpea showed both local and systemic symptoms. Disease progress was observed at different days post inoculation (dpi) till the death of the inoculated plants. –dsNSs, no dsNSs treatment was given prior to GBNV inoculation; +dsNSs, an amount of 5.0 μg/plant of dsNSs in autoclaved distilled water containing 0.01% Celite was applied followed by GBNV inoculation after 10–15 min (T2 treatment combination).

In semi qRT-PCR, of all the three combinations, the combination-T2 and -T3 showed a marked reduction of *NP* gene amplification compared to the control or the combination-T1 (Figure 2C). The qRT-PCR results with *NP* gene primers showed a significant reduction in the viral load in plants treated with dsRNA compared to the non-treated plants (Figure 2D). The maximum reduction of viral load (12.5 fold) was found in the treatment combination-T2: dsNSs + GBNV followed by the treatment combination-T3 and i, which were 3.57 and 1.81 fold, respectively (Figure 2D). In the treatment combination-T2, which showed the maximum reduction of the viral load, the plants survived for a longer period of time compared to the non-treated ones (Figure 4A). In the three independent experiments of three application combinations of dsRNA, plants survived upto 23–25 dpi, indicating an 8–10 days delay in the death of the plants due to GBNV infection.

### Efficiency of dsNSs Against Groundnut Bud Necrosis Virus in Cowpea

The efficacy of dsNSs was further examined in cowpea cv. Pusa Komal plants (*n* = 4, 10, and 3 plants in the three experiments). The primary leaves of cowpea cv. Pusa Komal inoculated with GBNV exhibited the chlorotic lesions at 4–6 dpi followed by necrotic lesions (Figure 3A), which coalesced, the inoculated leaves wilted and fell by 8–12 dpi. Subsequently, the plants developed systemic symptoms like necrotic lesions on newly developed leaves and necrosis on the petiole and stem. Eventually, the plants collapsed and died by 14–16 dpi. To determine the disease severity index, the level of the disease that is no-symptom to local necrosis and subsequent systemic necrosis followed by the death of plants were graded from 0 to 8 point scale (Table 1). The three treatment combinations of dsNSs and challenge with GBNV as carried out for *N. benthamiana* were also examined for cowpea. The dsNSs application on the two primary leaves of each seedling (5.0 μg/plant) in all the three treatments showed a significant reduction in the number of local lesions in cowpea seedlings (Figure 3D). The treatment combination-T2: dsNSs + GBNV showed a significant reduction of local lesions (1–2/seedling) compared to the non-treated plants (15–25 lesions/plant) (Figure 3A). The treatment combination T2 and T3 also resulted in a significant reduction of the number of lesions (3–5 lesions/plant) (Figures 3A,D). The dsRNA treatment combinations showed a significant effect on the reduction of local symptoms, which had a great implication on the restriction of *in planta* spread of the virus and survival of plant as when the non-treated plants died by 15–16 dpi due to the combined effect of local and systemic disease pressure, the dsRNA-treated plant survived with the development of new leaves (Figure 4B). The DSI, as well as AUDSC at 6 dpi in cowpea, were significantly low (DSI: 9.7–84.4 versus 100; AUDSC: 86.6–427.4 versus 544.1) in all the three combinations of dsRNA treatment as compared to the non-treated control (Table 2). The least DSI and AUDSC were obtained from the treatment combination-T2: dsNSs + GBNV. The presence of GBNV was detected by RT-PCR in all the dsRNA treatment combinations following the virus inoculation; however, the intensity of amplification was relatively less compared to the control especially in the case of the treatment combination T2 and T3 (Figure 3C). The GBNV load in the dsRNA-treated plants as judged by qRT-PCR was also significantly less compared to the non-treated control plants (Figure 3E).

## Discussion

In this study, we attempted to limit GBNV infection through the external application of dsRNA derived from the full-length gene of *NSs*. Our results indicate that the foliar treatment of plant with dsNSs significantly reduced the symptom expression as well as the viral load in the two experimental plant species, *N. benthamiana* and *V. unguiculata*.

RNA interference is an important mode of cellular immunity against viruses in the plant. The dsRNA plays a critical role in initiating RNAi in a cell. The preparation of dsRNA is an important step in the topical application approach of inducing RNAi. The dsRNA can be produced *in vivo* through bacterial expression or *in vitro* using RNA polymerase (Paula et al., 2012; Voloudakis et al., 2015; Papic et al., 2018; Li and Zamore, 2019). Different bacterial expression systems for obtaining dsRNA are known, which are more convenient and economic (Voloudakis et al., 2015). In the present study, we used the bacterial expression system to prepare dsRNA of *NSs* gene of GBNV, which is 1.3 kb long and is located toward the 5′ end of the S RNA genome segment of GBNV. Optimization of IPTG concentration, induction time, and different enzyme treatment resulted in a yield of about 1.5–2.0 mg dsNSs RNA per liter of bacterial culture. The yield of dsNSs was 50% less compared to that obtained in the published protocol (Posiri et al., 2013). This may be due to the difference in the length of dsRNA expressed in the two studies. Posiri et al., 2013 used dsRNA with a maximum size of 400 bp, whereas in the present study, the length of NSs dsRNA was more than three times longer.

In the external application of dsRNA, entry of dsRNA is imperative in the induction of RNAi. In plants, due to the presence of several physical barriers like wax layer, cuticle, cell wall, and cell membrane, delivery of dsRNA into the plant cell is a challenge (Bennett et al., 2020). Several methods like mechanical rubbing, pressure spray, infiltration, injection, root, or petiole absorption, nano-carrier conjugation of dsRNA have been used to deliver dsRNA into the plant cells for silencing of endogenous genes or plant virus genes (Tenllado and Diaz-Ruiz, 2002; Numata et al., 2014; Mitter et al., 2017; Dalakouras et al., 2018; Dubrovina and Kiselev, 2019). In the present study, dsNSs RNA aqueous suspension containing Celite was applied on the adaxial surface of leaves through gentle rubbing. Celite is known as diatomaceous earth and is used as an abrasive for the mechanical sap transmission of plant viruses (Hull, 2013). Our results showed that Celite could also facilitate the successful entry of dsNSs in the plant tissues of both *N. benthamiana* and cowpea. The RT-PCR detection of dsRNA both in local as well as distal leaves indicated the systemic movement of dsRNA within the plant system.

*Nicotiana benthamiana* plant is a super susceptible host of GBNV (Mandal et al., 2012). The inoculated leaves of *N. benthamiana* did not produce any local lesions as found in cowpea, rather they showed mild yellow blotches followed by rapid and severe systemic disease response. Hence, cowpea and *N. benthamiana* plants provided both local lesion and systemic assay systems for the evaluation of the efficacy of externally applied dsRNA against important tospovirus like GBNV. In the natural conditions, virus infection can occur any time before, during or after the application of dsRNA. Considering these possibilities, in the present study, dsNSs was applied on the leaves a day before (T1), immediately (T2), and a day after (T3) the challenge-inoculation of plants with GBNV. The analysis of disease progress showed lower DSI and AUDPC in dsRNA-treated plants of both cowpea and *N. benthamiana* and a strong positive correlation was established between DSI and AUDPSC values, which indicated a good covenant between the two parameters of disease measurement (Roy et al., 2009 and Chattopadhyay et al., 2010).

All the three dsRNA treatment combinations not only showed a significant reduction of disease severity, but also reduced the GBNV titer in both *N. benthamiana* and cowpea. The treatment combinations-T2 and -T3 appeared to be more effective compared to T1. The possible hypothetical explanation of such differential treatment effect may be, in T1, when dsNSs was applied 1 day prior to challenge inoculation, the resultant siRNA after biogenesis, might not immediately form an activated RISC complex in the absence of corresponding target transcript of *NSs* from GBNV. Hence, some portion of siRNAs might have been lost due to nucleolytic degradation (Ji and Chen, 2012) that might resulted in less efficacy. On the contrary, in other two treatments (T2 and T3), the presence of GBNV during or prior to the application of dsNSs might have immediately activated the RISC complex, resulting in minimal or no loss of siRNA; hence, the viral titer in these two treatments was lower than the first treatment. The lower viral titer in treatment T2 and T3 compared to T1 presumably contributed less symptom severity. Furthermore, GBNV infection during or prior to the dsNSs application might have triggered biogenesis of GBNV genome-wide siRNA and that might have further accentuated by the dsNSs treatment. However, such hypotheses need to be examined.

Induction of resistance through foliar application of dsRNA has been studied recently for TSWV, the type member of the genus *Orthotospovirus* (Tabein et al., 2020; Konakalla et al., 2021). The dsRNAs in these studies were derived from the partial sequence of *N, NSs*, and *NSm* genes of TSWV. It was found that the dsRNA obtained from these genes had differential efficacy. *NSs* gene of TSWV is known to act as a PTGS suppressor and plays a vital role in the proliferation of virus by interfering with the RNAi mechanism of the host (Takeda et al., 2002). The dsRNA derived from the 646 nt sequence of *NSs* gene of TSWV showed a lower level of resistance compared to that of dsRNA from the 717 nt sequence of *N* gene (Konakalla et al., 2021). However, the efficacy of full-length *NSs* is not known. NSs protein of GBNV was shown as a pathogenic factor as it induced necrosis symptom and plant cell death (Goswami et al., 2012; Singh et al., 2017). In this study, *NSs* being a symptom determinant, the dsRNA derived from the full-length gene was highly effective in protecting from GBNV infection in cowpea as well as in *N. benthamiana* plants. We chose to utilize the full-length NSs dsRNA as it would include all the effective siRNA (Gago-Zachert et al., 2019). Furthermore, the use of full-length dsNSs RNA may result in silencing expansion beyond the 5′ and 3′ regions of the NSs open reading frame (ORF) as the NSs transcript is longer than its ORF. The use of longer dsRNA compared to the smaller one is supposed to include bigger pool of siRNAs even after the expansion of silencing. The protective efficacy of dsNSs was observed superior in the case of cowpea compared to *N. benthamiana*. This may be due to a higher level of vulnerability of *N. benthamiana* to viral infection as it lacks RNAi factors to resist virus infection. Previous studies have shown that *N. benthamiana* did not possess an active salicylic acid- and virus-inducible RDR and hence it was hyper susceptible to viruses (Yang et al., 2004). This was further supported by another study showing that the natural loss of variant of RDR1 in *N. benthamiana* resulted in the hypersensitivity of this plant to a large number of viruses (Ying et al., 2010).

Although there was a reduction of disease progression and severity, the RT-PCR test showed the presence of GBNV in each inoculated plants. However, the load of GBNV as judged by semi qRT-PCR and qRT-PCR showed a significant reduction in the dsRNA-treated plants of both *N. benthamiana* (12.5 folds reduction) and cowpea (20 folds reduction). Though the single application of dsRNA showed protection for a limited time in *N. benthamiana*, all the inoculated plants were affected and eventually died. However, the majority of cowpea plants that were treated with dsRNA survived with the single application of dsRNA. The disease response of GBNV in *N. benthamiana* is drastic compared to cowpea. In transgenic plants, dsRNA is abundant in the plant system as it is continuously and synchronously generated in all the cells (Smith et al., 2000). In contrast, exogenous application of dsRNA contributes only a limited amount of dsRNA in the plant system (Konakalla et al., 2016; Kaldis et al., 2018; Namgiala et al., 2019). Therefore, effective delivery system and continuous application of dsRNA are required for the sustainable protection of plants from viral infection.

## Data Availability Statement

The original contributions presented in the study are included in the article/supplementary material, further inquiries can be directed to the corresponding author/s.

## Author Contributions

BM, SM, and AR planned the experimental layout. DG and OS performed the experiments. DG and BM wrote the manuscript. BM, SM, AR, and YB helped the trouble shooting for technical problems and revised the manuscript. All authors contributed to the article and approved the submitted version.

## Conflict of Interest

The authors declare that the research was conducted in the absence of any commercial or financial relationships that could be construed as a potential conflict of interest.

## Publisher’s Note

All claims expressed in this article are solely those of the authors and do not necessarily represent those of their affiliated organizations, or those of the publisher, the editors and the reviewers. Any product that may be evaluated in this article, or claim that may be made by its manufacturer, is not guaranteed or endorsed by the publisher.

## Footnotes

1
https://talk.ictvonline.org/taxonomy/

2
https://www.addgene.org/1654/

3
https://www.thermofisher.com
